# Dynamic Seat Assessment for Enabled Restlessness of Children with Learning Difficulties

**DOI:** 10.3390/s22093170

**Published:** 2022-04-21

**Authors:** Valentina Stanić, Taja Žnidarič, Grega Repovš, Gregor Geršak

**Affiliations:** 1 Faculty of Electrical Engineering, University of Ljubljana, 1000 Ljubljana, Slovenia; gregor.gersak@fe.uni-lj.si; 2 Department of Psychology, Faculty of Arts, University of Ljubljana, 1000 Ljubljana, Slovenia; taja.znidaric@ir-rs.si (T.Ž.); grega.repovs@ff.uni-lj.si (G.R.)

**Keywords:** ADHD, electrodermal activity, motion, cognition, facial temperature, thermal imaging, inertial measurement unit, dynamic seat, therapy ball

## Abstract

Children with Attention-Deficit/Hyperactivity Disorder (ADHD) face a range of learning difficulties in the school environment, thus several strategies have been developed to enhance or optimise their performance in school. One possible way is to actively enable appropriate restlessness using dynamic seats. In this paper, an assessment of the efficacy of a dynamic seat while solving school task is presented and compared to classic chair and therapy ball. To test the effectiveness of active seat, a study that examined task solving performance while observing the intensity of movement, in-seat behaviour and psychophysiological responses (electrodermal activity, facial temperature) was designed. A total of 23 school-aged children participated in the study, 11 children with a combined type of ADHD and 12 children without disorders. Children with ADHD achieved the best results when sitting in the active seat, where the most intense movement and best in-seat behaviour was observed. At the same time, psychophysiological parameters indicate that when performing better at the task children with ADHD were not too challenged and were consequently less agitated. Results have suggested that for a better cognitive performance of children with ADHD, it is crucial to provide a comfortable and pleasant workspace that enables them the right amount of restlessness.

## 1. Introduction

Neurological developmental disorder Attention-Deficit/Hyperactivity Disorder (ADHD) is expressed in children as difficulty maintaining attention, inhibiting behaviour (impulsivity), and managing excessive motor and speech activity [1,2]. Start of schooling coincides with the period of typically the most intense symptoms of ADHD [3]. At the same time, most diagnoses of ADHD are made during the early school years due to the occurrence of learning difficulties and disruptive behaviour during class [4].

ADHD is denoted with weaker executive functions such as inhibition, working memory, planning, and task switching [5,6,7]. Problems with reading and reading comprehension in children with ADHD are also explained by the higher incidence of specific learning disabilities [8], with as many as 25–40% of children with ADHD meeting the criteria for dyslexia [9]. In the school environment, this manifests itself as carelessness at school tasks, disorganization, interrupting the teacher (and classmates), poor listening and following instructions, speaking without permission, avoiding mentally demanding tasks, forgetfulness, restlessness, and poor in-seat behaviour [10,11]. Although all children occasionally exhibit such behaviour, it is so common and intense in children with ADHD that it prevents them from functioning normally in the school environment and in everyday life [2].

It is recommended to combine several measures to manage behaviour in children with ADHD at school, such as adjusted classroom arrangement (grid arrangement of desks, sitting close to the teacher and away from windows, lower lighting, larger workspace for a child), smaller classes, interesting and short explanations (treatment of material in small sets with regular breaks, multi-sensory presentations, e.g., use of visual aids, enrichment of the lesson with novelties, e.g., quizzes and films), sensible distribution and composition of tasks (shorter and original tasks, start of the class with easier tasks, which become more difficult over time), encouraging and rewarding good work habits (writing notes, daily report cards, raising the hand before talking), and preventing inappropriate behavior with a calm but firm warning [10,11,12,13].

Problems with the regulation of psychological arousal are also often attributed to ADHD [14]. Psychological arousal encompasses behavioural and physiological mechanisms for regulating the state of mindfulness and attention [14]. According to Yerkes–Dodson law [15], good cognitive functioning requires an optimal level of psychological arousal, determined by interactions between the central and autonomic nervous systems. To better understand the atypical functioning of the autonomic nervous system and the cognitive activity of children with ADHD, physiological parameters such as electrodermal activity and skin temperature can be measured.

Electrodermal activity (EDA) can be observed by measuring changes in the electrical properties of the skin due to the activity of the sweat glands [16,17]. Although the main function of sweating is the body thermoregulation, sweat glands are active also during psychological or emotional arousal and stressful situations [16,18]. Using the EDA signal, we can determine the level of psychological arousal by observing skin conductance level (SCL) and skin conductance response (SCR). SCL determines the level of psychological arousal and the baseline [16]. Current skin conduction values are described by SCRs, the density of which indicates the degree of psychological arousal (for example, more than 20 SCRs/min means high psychological arousal) [16]. SCRs represent values that exceed a certain threshold within the selected time frame [16]. Usually, SCR occurs 2–9 s after the start of the stimulus, when the amplitude of the SCR signal exceeds a threshold with a typical value between 0.01 µS and 0.05 µS [16]. The type of electrodes and the place of their installation depend on the purpose of the measuring device. The highest density of sweat glands is on the palms [16], so silver wet electrodes with a thin silver/silver chloride (Ag/AgCl) layer placed on the posterior joints of the index finger and middle finger are usually used [16,17]. A gel containing the electrolyte is required for optimal operation of such electrodes [16,17]. In the case of wearable sensors, the emphasis is on ergonomic design and ease of use, so dry stainless steel electrodes mounted on the upper arm are often used [17]. This site is suitable for measuring EDA, as all sweat glands are active in psychological sweating, and differences in the amount of sweat occur only due to the density of their distribution [19].

Body temperature depends on environmental conditions, both on biological conditions (adaptation to ambient temperature, overcoming a virus) and on emotional responses (social interactions, fight-or-flight) [20]. In the latter, the autonomic nervous system controls temperature through narrowing (vasoconstriction) and widening of subcutaneous vessels (vasodilation) and psychological sweating [20,21]. Vasoconstriction of peripheral facial vessels and increased cognitive load redirects blood flow from the face to the brain and thus affects the temperature image of the entire face [22,23]. The most stable temperature is in the forehead area and the most variable at the tip of the nose; therefore, these areas are often the subject of research [24,25,26]. There is also a very dense distribution of sweat glands on the forehead, so in case of stressful situations, psychological sweating is noticeable [19], which (negatively) affects the measurement of forehead temperature in the case of water film formation [27].

### Enabling Restlessness in School

Hyperactivity is a unique feature of ADHD [28], but its role is not entirely clear. One explanation is given by the theory of optimal stimulation [29], according to which hyperactivity is a mechanism that compensates for the lack of psychological arousal with an additional visual and kinesthetic contribution. Thus, increased activity occurs only in low-stimulation environments [29]. This theory is also supported by the model of functional working memory [30], which attributes to hyperactivity the role of stimulating the activity of the prefrontal cortex in demanding cognitive tests. At the same time, increased activity enables the avoidance of environmental requirements or tasks that are too demanding and overloading for their less developed working memory [30]. The model is supported by more research showing that children with ADHD move more intensely than typically developed children in more cognitively demanding tasks involving working memory load [31,32,33]. In contrast, in children without ADHD, increased exercise results in poorer functioning of their working memory [33]. Increased movement in children with ADHD may reflect the use of multiple cognitive resources [31]. With more intense physical activity, it is possible to strengthen the functioning of the cognitive control in children with ADHD [33].

The hyperactivity characteristic of children with ADHD therefore plays a functional role in their neurocognitive functioning [30,31,32,33]. The ability to direct and shift attention plays a key role in controlling movement [34]. Movement control is more effective when the individual has as many sources of attention as possible and at the same time as few distractions as possible [35]. Children with ADHD have shorter attention span [36]; therefore, movement control, imposed by any seating that does not allow spontaneous movement, can be an important consumer of attention. The positive link between hyperactivity, appropriate behaviour and problem-solving efficiency is also supported by other research [31,33], resulting in an idea of allowing for restlessness during class. Possible strategies are the use of dynamic seats (therapy ball, balance pillow, one-legged chair, standing desks), inclusion of physical activity in lessons (active games, moving furniture, carrying books, distributing papers to classmates, cleaning the board), holding classes outside and using classroom-friendly fidget toys [10,12,13].

Increasing physical activity and allowing for restlessness during class can also reduce the risk of many health problems resulting from prolonged sedentary position in school [37,38,39,40,41,42]. A commonly used alternative to the standard school chair is the therapy ball. Research on the impact of sitting on a therapy ball has divided opinions. While some confirm the positive effects of the therapy ball on in-seat behaviour and improving attention span in children with ADHD [43,44,45], recent research has not seen improvements in behaviour and productivity [46,47]. The potential benefits of the therapy ball have most likely not been revealed because it allows too much movement and overloads children with ADHD who have difficulties with self-control [47]. Despite the disagreement about the impact of the therapy ball on behaviour, there is no doubt about its popularity among children, as they labelled it as very comfortable and that it helped them improve concentration in solving tasks [43,46]. There are also many other kinaesthetic seats available that encourage active sitting in a unique way. This study evaluates an active seat that does not restrict legs movement and at the same time stabilizes the torso, thus enabling writing and solving tasks despite increased activity. The chair’s advantage is also in the adjustable height and shape of the seat, so it can be perfectly adapted to the child. This eliminates many of the potential negative consequences of an oversized or undersized seat [48,49,50]. This chair is still in the prototype phase, so it is necessary to check its actual effect on the cognitive performance of children with ADHD. To our knowledge, this study represents the first investigation of children’s cognitive performance using a series of cognitive tasks with simultaneous observation of the observed child’s movement and psychophysiology.

## 2. Materials and Methods

To comprehensively address the impact of seating type on student performance, an experiment was conducted that focused on cognitive tests, observation of movement and psychophysiological response of children. The study examined the effectiveness of an active seat, a therapy ball, and a standard school chair. The main focus was on whether changing the seat could improve the performance of children with ADHD in solving school assignments, what would be the most appropriate solution for children with ADHD in the school environment and whether the change of seat in any way also affects the effectiveness of children without ADHD.

### 2.1. Participants

The study included 23 children, 11 children with a combined type of ADHD (8 boys and 3 girls aged between 9 and 11) and 12 children without ADHD (6 boys and 6 girls aged between 8 and 10). Children had to be medication-free for at least 24 h before the start of the experiment. Children with ADHD also had various comorbid disorders listed in Table 1. The parents or legal representatives of all children signed a consent form to participate in the research, which was approved by the Ethics Committee of the Faculty of Arts, University of Ljubljana (application number: 167-2019).

### 2.2. Instrumentation

#### 2.2.1. Seats

**Figure 1:**
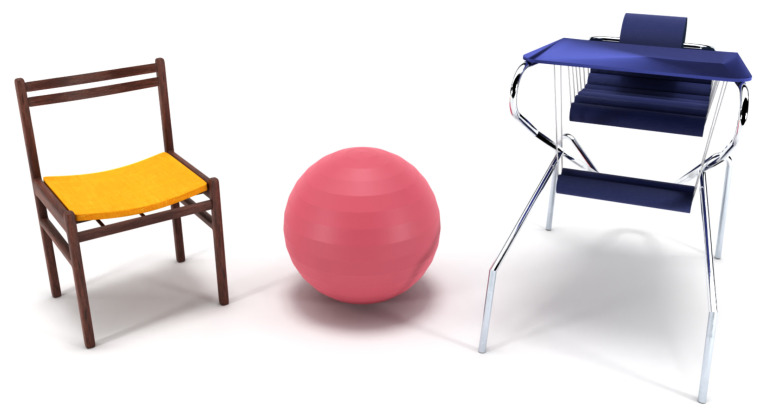
From left to right: school chair, therapy ball, active seat.

Three seats (Figure 1) were used in the experiment: a classic school chair with a typical design—a wooden frame with a backrest and no armrests, a standard therapy ball of two sizes (ball diameter: 45 or 55 cm), where the appropriate size was chosen according to the height of the participant, and an active seat. The design of the latter consists of a metal frame with a flexible seat and backrest as well as an adjustable footrest that mimics a swing. The seat, backrest and footrest are softly padded. Depending on the height of the individual, the seat is raised and adjusted enabling feet to move freely. The chair also has armrests and a removable plate that substitutes the desk.

#### 2.2.2. Inertial Measurement Unit

**Figure 2:**
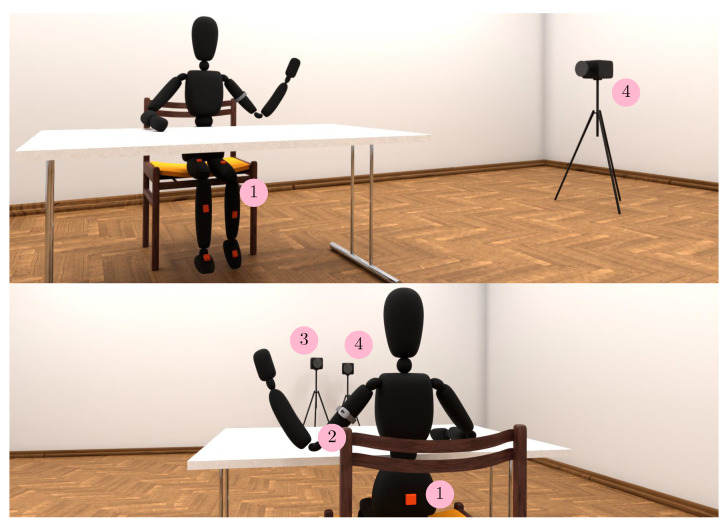
Measuring system with measuring instruments: 1—IMU, 2—SenseWear sensor, 3—thermal imaging camera, 4—(RGB) video camera.

The intensity of movement of the lower limbs was measured using accelerometers in seven inertial measurement units (IMU), which were placed on the instep of the left and right foot, the front of the left and right shank, the front of the left and right thigh, and the sacrum (Figure 2). The estimated attachment error was 2 cm. The measuring range of the accelerometer was set to 2 g. The sensor part of each IMU consists of a three-axial accelerometer STM LIS331DLH, a three-axial gyroscope Invernsense IMU-3000 and a three-axial magnetometer Honeywell HMC5883 [51]. IMU has a built-in 3.7 V battery with a capacity of 250 mAh, microcontroller and transmitter. The used user interface was built in the MATLAB/Simulink R2019b environment to capture and transfer IMU data [52]. IMU signals were sampled at a frequency of 80 Hz.

#### 2.2.3. SenseWear Sensor

To measure skin conductance level, the BodyMedia SenseWear AB155 was used, which was placed on the upper arm of a non-dominant hand, as shown in Figure 2. The device does not have a power button, but turns on automatically within 10 min of skin contact. To ensure the operation of the device during the tests, it is necessary to dedicate 10–15 min to the preparations [53]. The sampling frequency of the skin conductance was set to 32 Hz, and for energy consumption 1 Hz.

#### 2.2.4. Thermal Images and Cameras

Facial skin surface temperature was monitored using the FLIR T650sc thermal imaging camera, which captures 24 bit images with a thermal sensor resolution of 640 × 480 with 50 fps. The emissivity factor was set to 0.98 and the distance to 3 m (Figure 2). Two video cameras, the Sony HXR-MC50E and the Sony Handycam HDR-CX6, were also part of the measurement system. The first captured the side view of the participant and the second the front view (Figure 2), together providing a complete overview of the participant’s movement.

#### 2.2.5. Cognitive Tests

To observe (allegedly) weaker executive functions three equivalent sets of five cognitive tests were prepared.

The estimated capacity of the spatial working memory (SWM) [54] was measured by a computer test in the PsychoPy 1.90.3 software. Participants had to renew the sequence of coloured squares that appeared on the screen, where the sequence lengthened with each attempt. The task was terminated after two incorrect answers. The number of correct answers represented the estimated capacity of the SWM.

Estimated verbal working memory (VWM) capacity [54] was assessed with three variants of the numerical range: numerical range forward (participant repeats the heard string of numbers in the same sequence, e.g., 3, 2, 6 renews as 3, 2, 6), numerical range backwards (participant restores the heard string of numbers in reverse order, from last to first, e.g., 3, 2, 6 renews as 6, 2, 3) and editing the numeric range by size (participant edits the heard string of numbers from smallest to largest, e.g., 3, 2, 6 renews as 2, 3, 6). The shortest string consisted of three numbers, and with every correct answer, the string lengthened. In case of a wrong answer, another string of the same length was given. If the latter could not be restored, the task was terminated. Numbers 0–9 were included. The average number of correct answers of all three variants represented the estimated capacity of the VWM.

The ability to switch tasks was assessed using the adapted Trail Making Test (TMT-AD) [55]. Participants had to correctly connect the circled numbers and letters, without lifting the pen from the desk and crossing links with each other. The TMT-AD consisted of three parts: The first part included circles with numbers 1–25. Participants had to connect the circles in ascending order (1, 2, 3, 4, …, 25). The second part included circles with numbers 1–12 and letters A-L. Participants had to connect the circles in ascending (numerical and alphabetical) order, while exchanging numbers and letters (1, A, 2, B, 3, C, 4, D, …, 12, L). The third part consisted of empty circles, connected with marked links. The participant had to connect the circles by following the links. Participants were able to correct any errors and proceed with the task solving, which only extended the overall time. The average solving time of all three parts was chosen for the representative value.

The Reading Comprehension (RC) task consisted of text and questions about the read text. The tasks were taken from the national test of Slovene langugage for the 6th grade of primary school. As participants belonged to a younger age group, they could ask the researcher for an explanation in case of incomprehension of the instructions or words. RC score was the proportion of correct answers.

The planning capabilities were evaluated using an adapted computer version of the Tower of London test (ToL-AD) [56]. The share of correct estimates of the participant to get the required arrangement of balls in a figure with the smallest number of moves was considered. The task consisted of 20 cases where 2 min was available for each.

### 2.3. Measures

#### 2.3.1. Movement Intensity

To measure the movement intensity of the participants, seven IMU with a built-in accelerometer that measures accelerations in all three axes of the local coordinate system were used. The intensity of the movement is represented by the dynamic component of the acceleration, so the contribution of gravity was mathematically removed.

#### 2.3.2. In-Seat Behaviour

The in- and out-of-seat behaviour was observed. In this study, the in-seat behaviour is defined as proper, when it did not hinder writing and solving tasks, as it allowed for less torso activity. The main information was determined by the IMU on the sacrum (${\mathrm{IMU}}_{1}$), because the intensity of torso movement can be inferred directly through measurements. For example, a child who heavily bounces on the ball (high ${\mathrm{IMU}}_{1}$ value) finds it very difficult to write. In addition, the average of the representative IMU values on both legs (from ${\mathrm{IMU}}_{2}$ to ${\mathrm{IMU}}_{7}$) was calculated and compared to the ${\mathrm{IMU}}_{1}$ value. Higher leg activity in comparison to the sacrum can be interpreted as a more proper in-seat behaviour.

#### 2.3.3. Cognitive Tests Solving Performance

With specific cognitive tests from Section 2.2.5 the estimated capacity of spatial and verbal working memory, the ability to switch tasks, reading comprehension and planning skills were assessed.

#### 2.3.4. Electrodermal Activity

Psychological arousal of children was observed by measuring skin conductance. The average of measurements within each cognitive test was calculated, which can be thought of as the skin conductance level (SCL) of the test in question. Electrodermal activity is represented by the relative skin conductance (SC-R), which is determined by the ratio between the average of all measurements of a particular test and the baseline. The latter is defined as an average of all measurements during 1 min resting period, ending 2 min before the beginning of the first cognitive test. SC-R can also be interpreted as a deviation from the baseline, both in the positive and negative direction.

#### 2.3.5. Facial Temperature

**Figure 3:**
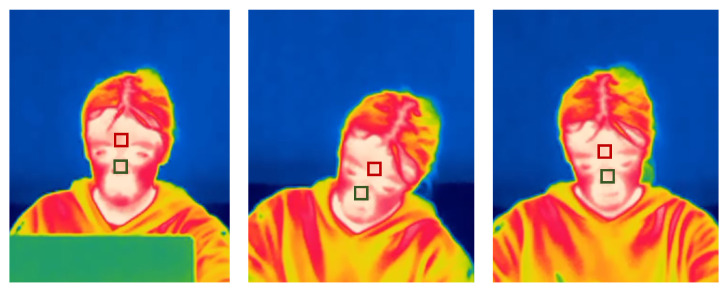
ROI on forehead (red frame) and nose (green frame) of one participant during different tasks.

Images of key measurement moments were extracted from the thermal imaging camera videos for each cognitive test, thus presenting temporal changes in temperature during the measurement. Similar to other research [24,25,26], the forehead and the tip of the nose were chosen as region of interest (ROI) as presented in Figure 3. The size of each ROI was 3 × 3 pixels. For each cognitive test, thermal images at key measurement moments were obtained: at the moment of the explanation of the task, as well at the beginning and the end of task solving.

#### 2.3.6. Subjective Assessment

Participants chose the most likeable seat and task as well as evaluated the intrusiveness of the measurement system. The latter answers were divided into three categories: *intrusive measurement system*—the participant was constantly aware of the measurement system and was also burdened by it, *less intrusive measurement system*—the participant was aware of the measurement system, but eventually got used to it and did not deal with it during the measurement, and *non-intrusive measurement system*—the participant completely forgot about the measurement system during the measurement.

#### 2.3.7. Statistical Analysis

In the study, participants sat in three seats; therefore, in addition to descriptive statistics, a two-way analysis of variance (ANOVA) for mixed plans, which evaluated the differences in intensity of movement, in-seat behaviour, performance at cognitive tests and SC-R values both between groups (children with and without ADHD) and within an individual group, was performed. The unrepeatable factor was Group (children with and without ADHD) and the repeatable factor was Seating (active seat, therapy ball, school chair). The limit value for statistical significance was set at p=0.05. Two-way ANOVA was not used at facial temperature due to deficient measurement values. For assessing differences between the children with and without ADHD in choosing the most likeable seat and task as well as the evaluation of the intrusiveness of the measurement system the Fisher’s exact test was used, where p≤0.05 determined statistically significant differences.

#### 2.3.8. Protocol

**Figure 4:**
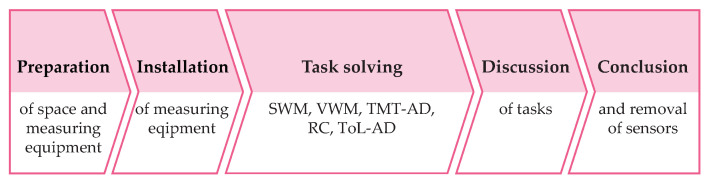
Measurement protocol.

In order to minimise the measurement anxiety of the ADHD participants, the experiment was performed at the counselling centre, where the participants with ADHD receive counselling and therapeutic services. The measuring protocol consisted of preparation for measurement, installation of measuring equipment, solving five cognitive tests, and a short discussion of tasks and measuring instrumentation. A simplified diagram of the measurement protocol is shown in Figure 4.

In designing the protocol and order of cognitive tests, the emphasis was on the exchange of more and less popular tasks in order to reduce the impact of the measurement on the result. The RC and the VWM test were evaluated as less pleasant; therefore, the cognitive tests were performed in the following order: SWM test, VWM test, TMT-AD, RC, and ToL-AD. For optimal efficiency of children, all measurements were performed in the morning, thus reducing the possibility of the occurrence of exciting events before the measurement, such as an exam or physical education class. To ensure constant illuminance, air temperature and humidity of the room the blinds were closed and lights turned on.

The movement and emotional state of the children were monitored with a sensory system that covered three areas of interest: intensity of movement, in-seat behaviour and psychophysiological responses. It was taken into consideration that children with ADHD have difficulty maintaining attention; therefore, highly intrusive measurement instrumentation was avoided [57].

The measurement began with greeting the participant and attaching the measuring equipment. To ensure the operation of the SenseWear AB155 sensor and the thermal adjustment of the facial temperature, 10–15 min were spent on the attaching of the measuring system and the preparation of the seat. First, the SenseWear AB155 sensor was atteched and then all the IMUs. Next, the seat was adjusted to the size of the participant. During the installation of the seat and measuring instruments, the researcher talked to the participant to relax them as much as possible and to decrease potential measurement anxiety. Afterwards, the participant began to solve cognitive tests, which were always in the same sequence. After each test, a short break (maximum 2 min) was possible at the request of the participant. At the end, a short conversation followed, where the participant assessed the perceived disturbance of the measuring system and the most likeable chair.

Each participant took three measurements, each time sitting on a different seat. The three seats allow for six combinations, so for ensuring the randomness of the experiment, the order of the seats was determined by classifying the children into six groups, as shown in Table 2. In each group, there were children with and without ADHD.

## 3. Results

**Figure 5:**
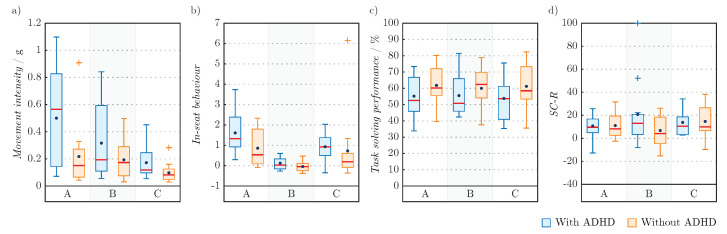
(**a**) Intensity of movement, (**b**) in-seat behaviour, (**c**) cognitive tests solving performance, (**d**) SC-R.

Results are presented with mean value (*M*) and standard deviation (*s*) as well as median value (*Me*). Statistical representation of movement intensity in all seats is shown in Figure 5. A higher value represents a higher intensity. The figure shows that the average movement intensity of children with ADHD is higher than children without ADHD regardless of the seat, with the highest scatter in the active seat.

Children with ADHD in all seats moved more intensely compared to the children without ADHD. On average, children with ADHD moved most intensively in the active seat (M=0.50 g, s=0.36 g, Me=0.57 g) and least in the school chair (M=0.17 g, s=0.12 g, Me=0.12 g). Children without ADHD were also, on average, most lively in the active seat (M=0.22 g, s=0.23 g), where the largest difference between children with and without ADHD was observed. Compared to the *Me* values, the smallest difference in activity between children with and without ADHD is on the therapy ball, where the *Me* of the children without ADHD is the highest (Me=0.17 g).

In-seat behaviour can have a negative value, which indicates a more active IMU on the sacrum compared to the average activity of all IMU on the legs and, consequently, less proper in-seat behaviour. The statistical representation of the in-seat behaviour in all seats is shown in Figure 5. The figure shows that children with and without ADHD exhibited the least proper in-seat behaviour while sitting on the therapy ball (children with ADHD: M=0.12, s=0.29, Me=0.03; children without ADHD: M=−0.04, s=0.24, Me=−0.05) and the most proper while sitting in the active seat (children with ADHD: M=1.61, s=0.96, Me=1.33; children without ADHD: M=0.86, s=0.86, Me=0.53).

The statistical representation of the cognitive tests solving performance on each chair is shown in Figure 5. Children with ADHD performed worse compared to the children without ADHD, regardless of the seat. On average, children with ADHD performed best while sitting on the therapy ball (M=55.5%, s=12.0%) and worst while sitting in the school chair (M=53.8%, s=13.0%). However, according to the median values, the opposite is true—children with ADHD achieved best results while sitting in the school chair (Me=53.6%) and worst results on the therapy ball (Me=50.8%). In addition to the achieved result, it is also beneficially to look at the time spent on solving the task. Table 3 shows that both children with and without ADHD in most cases spent more time on task solving while sitting in the active seat than on the therapy ball or school chair. At the same time, children with ADHD solved the VWM and RC tasks faster than children without ADHD.

The statistical representation of the electrodermal activity in all seats is shown in Figure 5. According to the results, the SC-R of children with ADHD was highest on the therapy ball (M=20.76, s=29.07, Me=13.05) and lowest in the active seat (M=10.78, s=10.44, Me=9.62).

**Figure 6:**
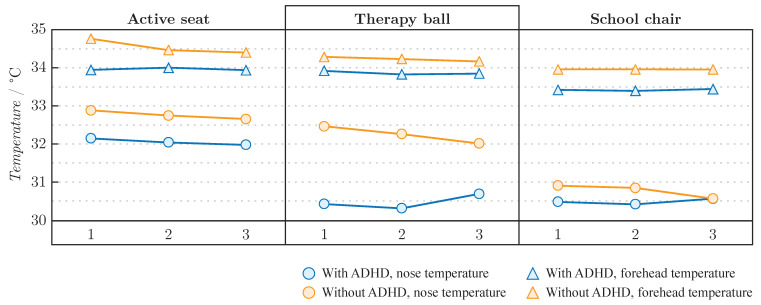
Average forehead and nose temperature of children with and without ADHD while solving cognitive test in active seat, therapy ball and school chair. Moments of interest are marked as 1—explanation of the task, 2—start of task solving, 3—end of task solving.

The average nose and forehead temperatures of children with and without ADHD are shown in Figure 6 and Table 4. The forehead temperature of children with and without ADHD was more stable compared to the temperature of the nose. The forehead and nose temperatures of children with ADHD were lower in all three seats compared to the temperatures of children without ADHD. Average forehead and nose temperatures of both children with and without ADHD were highest while sitting in the active seat and lowest in the school chair. The smallest difference between the forehead and nose temperatures of children with ADHD was on the active seat, while the most prominent difference was on the therapy ball.

Table 5 shows the results of the two-way ANOVA for mixed plans. It can be seen that the seat had a significant effect on movement intensity. Further analysis of simple contrasts revealed that participants were moving significantly more intense while sitting in the active seat compared to the therapy ball, $F\left(1,21\right)=5$.062, p=0.035, $\eta_{p}^{2}=0$.194, and the school chair, $F\left(1,21\right)=15$.490, p=0.001, $\eta_{p}^{2}=0$.425. Furthermore, simple contrasts analysis showed significant more intense movement on the therapy ball compared to the school chair, $F\left(1,21\right)=16$.162, p=0.001, $\eta_{p}^{2}=0$.435. Results in Table 5 also report significant effect of the seat on the in-seat behaviour of participants. Further simple contrasts analysis revealed significantly less proper in-seat behaviour on the therapy ball compared to the active seat, $F\left(1,21\right)=42$.922, p≤ 0.001, $\eta_{p}^{2}=0$.671, and the school chair, $F\left(1,21\right)=7$.200, p=0.014, $\eta_{p}^{2}=0$.255. No factor had a significant effect on test solving performance and electrodermal activity.

The most likeable cognitive test among children with ADHD after sitting in the active seat were SWM and ToL-AD (27.3%) and the least favourite RC (72.7%). Similarly, after sitting on the therapy ball children with ADHD most preffered SWM test (38.5%) and least RC (63.6%). After solving tasks on the school chair most children with ADHD chose ToL-AD as their favourite task (50.0%) and again RC as the least favourite task (72.7%). Fisher’s exact test revealed no significant difference between children with ADHD and children without ADHD in choosing the most and the least favourite task regardless of the seat.

Children with and without ADHD liked active seat the most (children with ADHD: 66.7%; children without ADHD: 75.0%) and school chair the least (children with ADHD: 0.0%; children without ADHD: 8.3%). The similarity of responses between children with and without ADHD was also confirmed by Fisher’s exact test, which showed that there was no significant difference in the seat preference among the participants (p=0.640).

None of the participants judged the measurement system to be intrusive. Fisher’s exact test showed that there were no significant differences between children with and without ADHD in the assessment of intrusiveness of the measuring system after measurement in the active seat (p=0.285), therapy ball (p=0.392), nor after measurement in the school chair (p=0.618).

## 4. Discussion

The exact cause of ADHD is not yet known, so several theories have been developed based on the atypical brain function and structure. As a result, there is no single solution to alleviate the symptoms of ADHD, but the measures depend on numerous factors, such as the age of the child and the intensity of the symptoms. The structured school environment uncover problems managing excessive physical activity and maintaining attention; therefore, teachers and parents try to help children with professional treatments, promoting positive work habits and a healthy lifestyle, adapting the workplace at home and school, implementing appropriate class structure as well as various strategies to encourage motor activity during learning and solving school tasks. The latter include the use of dynamic seats, such as a therapy ball and an active seat, which children usually like more than a standard school chair.

This study evaluated whether a change of seat improves the cognitive performance of children with the neurological developmental disorder ADHD by observing their movement patterns and psychophysiological responses when solving various cognitive tests. The results show that, on average, children with ADHD performed worse in all seats than children without ADHD, which is in line with generally accepted assumptions about impaired executive functions [5,58,59,60].

**Figure 7:**
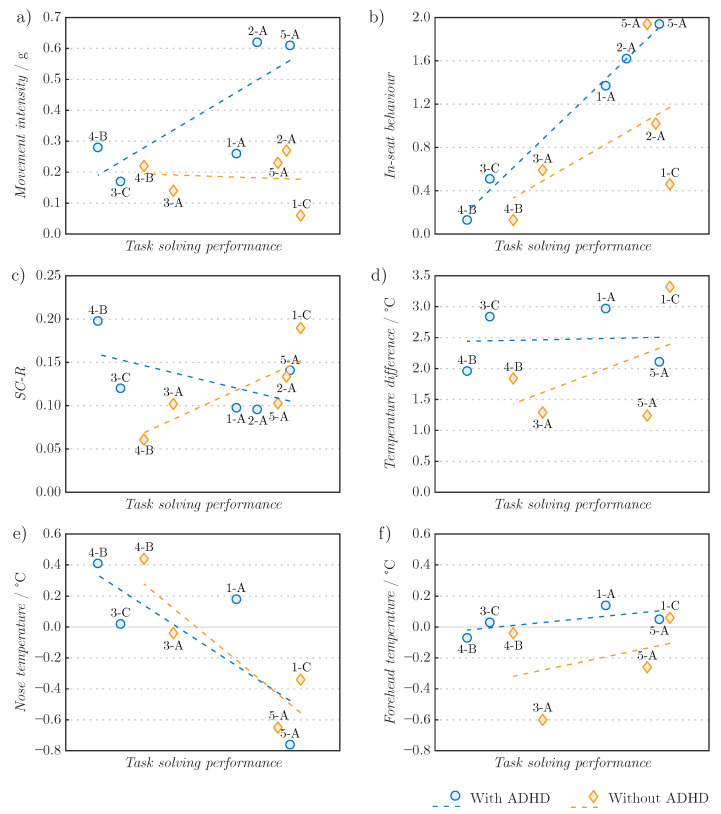
Average (**a**) movement intensity, (**b**) in-seat behaviour, (**c**) SC-R, (**d**) nose and forehead temperature difference, (**e**) nose temperature and (**f**) forehead temperature of children with and without ADHD while solving cognitive tests, with trendlines. The cognitive tests are marked as 1—SWM, 2—VWM, 3—TMT-AD, 4—RC and 5—ToL-AD. The seats are marked as A—active seat, B—therapy ball and C—school chair. For each cognitive test best average result with corresponding seat is presented (e.g., 4-B is best solved RC, which happened to be on the therapy ball).

As seen in Figure 7, children with ADHD were generally moving more intensively in all three seats, compared to the children without ADHD. Similar results are found in other studies [31,33] that regard hyperactivity in children with ADHD as a mechanism to compensate for low arousal and short attention span. This aspect is also confirmed by the theory of optimal stimulation [29] and the model of functional working memory [30], which associate the higher intensity of movement of children with ADHD with a more demanding cognitive tasks. Thus, we can conclude that in children with ADHD, more intense movement indicates a more successful test solving performance. Furthermore, in children with very intense excessive movement, it was observed that the active seat did not keep them in the workplace. This was most evident during the unpopular reading comprehension test, where hyperactive children calmed down only after standing up. Here, an advantage in the height of the work surface in the active seat was shown, as they could read and write without awkwardly leaning over the table. Standing desks are an interesting proposal for further studies, as they are adaptable and much easier to implement in classrooms for all children [61,62,63].

Studies [43,44,46,47] emphasize that with dynamic seats, it is not only the intensity that is important, but also the in-seat behaviour. Both children with and without ADHD exhibited better in-seat behaviour while achieving better results in cognitive tasks (Figure 7). Although the therapy ball allows for intense movement, the movement can be excessive and thus negatively affect effectiveness of the children in tackling cognitive tasks. This also explains why some research reports positive effects of the therapy ball on the behaviour and productivity of children with ADHD [43,44,45], while others have not seen any improvement [46,47]. Consequently, we can predict that in children with ADHD, more proper in-seat behaviour correlates with better performance on tests. It should be noted here that the improper in-seat behaviour characterise behaviour that hinders children from solving tasks and is disturbing to the surroundings (e.g., children frequently leaving the seat).

Figure 7 shows that in children with and without ADHD, the SC-R value increased with each test. In children with ADHD, the increase in mean SC-R was less pronounced with better performance on the test, while in children without ADHD, the opposite was observed. A higher SC-R level indicates a greater cognitive load [64], but in a more difficult test, the level of SC-R may be lower if the children perceive it as easy or boring [65] and if they were very impatient or enthusiastic during the explanation. Elevated SC-R levels are also characteristic of various emotional states such as anxiety, disgust, excitement, and joy [66]. Due to the wide range of probable emotions, it is not possible to properly distinguish between them by observing SC-R alone [67], so it is necessary to consider additional, even redundant parameter of the autonomic nervous system, such as facial skin temperature. Nevertheless, less intense and more subdued emotions are characterized by a less pronounced rise in SC-R levels [68]. Therefore, it can be concluded that children with ADHD were less agitated and (probably) less cognitively burdened during more successful task solving, while children without ADHD responded in exactly the opposite way.

Children with ADHD had similar average nasal tip temperature and higher average forehead temperature compared to children without ADHD (Figure 7). The nasal tip temperature for children with and for children without ADHD generally decreased with more successful test solving performance, while forehead temperature increased. The study [22] associates this type of facial temperature picture with a more demanding cognitive task. Cooling of the nose and warming of the forehead may indicate joy, stress, anger, or anxiety [20,66,69,70]. By considering the level of SC-R, a moderate feeling of anxiety or agitation can be attributed to the children with ADHD during the more successful test solving performance, and the children without ADHD were feeling more stressed. The forehead temperature was more stable compared to nasal temperature, which is consistent with other studies [24,26,71]. Based on the results of facial temperature, we can conclude that children with ADHD were more cognitively burdened and less agitated when they performed better on tests. From Figure 6, no prominent change in temperature between the explanation and the beginning of solving the task can also be observed. Therefore, it can be concluded that adequate explanation prepares children for the task and increase their engagement in the problem solving.

Subjective assessments report that children preferred the test where they achieved better results, and conversely, the test where they performed the worst caused them the most problems and was the least popular with them. In addition, the seat where they were more successful in task solving was more liked by all children irrespectful of ADHD.

Participants predominantly performed best in the active seat, which was also the most popular choice. Children with ADHD mostly achieved the worst results in the school chair, and children without ADHD on the therapy ball. Thus, we can conclude that active seat is the best choice for both groups of children, but its effects are not sufficiently pronounced to significantly contribute to better cognitive performance of children.

### Limitations

Certain limitations of the research also need to be addressed. The selected sample of children was not representative or large enough. Given that the intensity of ADHD symptoms may decrease with age, an age-homogeneous sample should be selected. It could also include only children without prescribed treatment with medications, thus excluding its potential impact on the cognitive performance and behaviour of children. Perhaps the effect of the active seat would be expressed in children with predominantly inattentive or hyperactive/impulsive type of ADHD disorder. To better understand the performance and behaviour of children, their school grades, IQ, and personality traits could also be considered.

The children were not bothered by the measuring system, so the choice of less intrusive and non-contact measuring devices was appropriate. Nevertheless, improvements to the measurement system are possible. The IMU signal could be used to calculate the kinematic model, thus obtaining accurate information on the trajectory of motion as well as the actual contribution of gravity. The thermographic camera could be closer to the face, as this would allow for more appropriate ROI determination. During the measurement, the children occasionally touched the face, which potentially affected the measured temperature. For a more reliable assessment of the psychological state, the sensory system could be supplemented by the temperature of the corners of the eyes and the measurement of an additional parameter regulated by the autonomic nervous system, such as heart rate.

## 5. Conclusions

Dynamic seats can be distinguished according to the movement they allow. Versatile and arbitrary movement is possible on an affordable therapeutic ball, but it can also be overwhelming. Children, especially those who are severely over-active, can be hindered from writing, listening, and completing school assignments by such a flexible seat. As a result, a number of dynamic seats that try to find a balance between allowing movement and ensuring stability while sitting are available on the market.

The study of enabling sedentary restlessness included cognitive tests to address performance in school children with and without ADHD, and the observation of movement intensity, in-seat behaviour, electrodermal activity, and facial temperature.

This study revealed best perfomance of children with ADHD in solving tasks in an active seat. In doing so, they moved very intensely with mostly proper in-seat behaviour, as they predominantly waved their legs and not the trunk, and accoriding to the psychophysiological parameters they were not too challenged or anxious about the task. The active seat failed to retain children with severe hyperactivity in the seat. However, the height of the workspace, similar to the standing desks, enabled children comfortable writing surface while standing and consequently help them to mantain attention on the task.

This research shows that active seat in these circumstances does not have a significant effect on the learning difficulties of children with ADHD, but it does have certain positive qualities that can be a starting point for further work. It turns out that children with ADHD need a comfortable and pleasant work space that allows them the right amount of restlessness, such as swinging their legs, squeezing a ball and standing while working. The research also sheds light on the problem of differences in intensity of the symptoms of ADHD, which makes it difficult to find a unique solution for the learning disabilities of children with this neurological disorder.

## Figures and Tables

**Table 1:** Comorbid disorders of children with ADHD.

Participant	Comorbid Disorders
ES01	/
ES02	Specific learning disabilities
ES03	Dyslexia
ES04	Dyslexia
ES05	Dyslexia
ES06	Specific learning disabilities
ES07	Speech and language disorder, Specific learning disabilities
ES08	Developmental co-ordination disorder (dyspraxia)
ES09	/
ES10	Dyslexia
ES11	Specific learning disabilities

**Table 2:** Seating order for participants with ADHD (ES) and without ADHD (KS). The seats are marked as A—active seat, B—therapy ball and C—school chair.

Seating Order	Participants
A-B-C	ES01, KS01, ES07, KS07
A-C-B	ES02, KS02, ES08, KS08
B-A-C	ES03, KS03, ES09, KS09
B-C-A	ES04, KS04, ES10, KS10
C-A-B	ES05, KS05, ES11, KS11
C-B-A	ES06, KS06, KS12

**Table 3:** Mean value of the task solving times for children with and without ADHD. The seats are marked as A—active seat, B—therapy ball and C—school chair.

		Children with ADHD [s]	Children without ADHD [s]
	A	88.0	92.1
SWM	B	93.5	88.9
	C	59.6	97.6
	A	193.3	224.5
VWM	B	177.3	206.0
	C	174.8	201.2
	A	189.8	136.1
TMT-AD	B	184.0	135.6
	C	153.9	130.4
	A	743.1	852.8
RC	B	707.3	825.7
	C	680.6	840.3
	A	315.0	261.2
ToL-AD	B	265.3	251.1
	C	256.4	245.1

**Table 4:** Average values of nose and forehead temperatures for children with and without ADHD in all three seats. The seats are marked as A—active seat, B—therapy ball and C—school chair.

		A [°C]	B [°C]	C [°C]
	Forehead	34.0	33.9	33.4
Children with ADHD	Nose	32.1	30.5	30.5
	Difference	1.9	3.4	2.9
	Forehead	34.5	34.2	34.0
Children without ADHD	Nose	32.8	32.2	30.8
	Difference	1.8	2.0	3.2

**Table 5:** Two-way ANOVA for factors Group and Seat.

Source of Variation	*SS*	*df*	*MS*	*F*	*p*	$\eta_{p}^{2}$
Movement Intensity						
Group	0.441	1	0.441	4.049	0.057	0.162
Error(Group)	2.286	21	0.109			
Seat	0.576	2	0.288	12.014	<0.001	0.364
Seat × Group	0.137	2	0.068	2.850	0.069	0.120
Error(Seat)	1.007	42	0.024			
In-seat Behaviour						
Group	2.396	1	2.396	1.688	0.208	0.074
Error(Group)	29.813	21	1.420			
Seat ^1^	16.899	1.600	10.562	11.838	<0.001	0.361
Seat × Group ^1^	1.213	1.600	0.758	0.850	0.414	0.039
Error(Seat) ^1^	29.977	33.600	0.892			
Cognitive Tests Solving Performance						
Group	656.628	1	656.628	1.587	0.222	0.070
Error(Group)	8688.643	21	413.745			
Seat	12.238	2	6.119	0.123	0.885	0.006
Seat × Group	27.126	2	13.563	0.273	0.763	0.013
Error(Seat)	2088.831	42	49.734			
Electrodermal Activity						
Group	565.688	1	565.688	1.622	0.218	0.079
Error(Group)	6628.284	19	348.857			
Seat	93.566	2	46.783	0.190	0.828	0.010
Seat × Group	742.876	2	371.438	1.505	0.235	0.073
Error(Seat)	9378.676	38	246.807			

^1^ Greenhouse-Geisser correction.

## Data Availability

The data presented in this study are available on request from the corresponding author.
